# Developing a predictive model and uncovering immune influences on prognosis for brain metastasis from lung carcinomas

**DOI:** 10.3389/fonc.2025.1554242

**Published:** 2025-03-03

**Authors:** Bowen Wang, Mengjia Peng, Yan Li, Jinhang Gao, Tao Chang

**Affiliations:** ^1^ Department of Gastroenterology, West China Hospital, Sichuan University, Chengdu, China; ^2^ Department of Emergency, General Hospital of Tibet Military Command, Lhasa, China; ^3^ Physical Examination Center, General Hospital of Western Theater Command, Chengdu, China; ^4^ Department of Neurosurgery, West China Hospital, Sichuan University, Chengdu, China

**Keywords:** lung carcinoma, brain metastasis, prognosis, model, immunology

## Abstract

**Objective:**

Primary lung carcinomas (LCs) often metastasize to the brain, resulting in a grim prognosis for affected individuals. This population-based study aimed to investigate their survival period and immune status, while also establishing a predictive model.

**Methods:**

The records of 86,763 primary LCs from the Surveillance, Epidemiology, and End Results (SEER) database were extracted, including 15,180 cases with brain metastasis (BM) and 71,583 without BM. Univariate and multivariate Cox regression were employed to construct a prediction model. Multiple machine learning methods were applied to validate the model. Flow cytometry and ELISA were used to explore the immune status in a real-world cohort.

**Results:**

The research findings revealed a 17.49% prevalence of BM from LCs, with a median survival of 8 months, compared with 16 months for their counterparts (*p <*0.001). A nomogram was developed to predict survival at 1, 3, and 5 years on the basis of these variables, with the time-dependent area under the curve (AUC) of 0.857, 0.814, and 0.786, respectively. Moreover, several machine learning approaches have further verified the reliability of this model’s performance. Flow cytometry and ELISA analysis suggested the prediction model was related the immune status.

**Conclusions:**

BM from LCs have an inferior prognosis. Considering the substantial impact of these factors, the nomogram model is a valuable tool for guiding clinical decision-making in managing patients with this condition.

## Introduction

Lung carcinomas (LCs) constitute a significant global burden with respect to carcinoma-related mortality (1). In the United States, it is projected that over 245,700 deaths will be attributed to LCs by 2025, accounting for more than a quarter of all carcinoma-related deaths (2). Similarly, China reported approximately 631,000 deaths related to LCs in 2015, with a crude mortality of 45.87/100,000 individuals (3). Non-small cell lung cancer (NSCLC), comprising squamous cell carcinoma and adenocarcinoma, accounts for more than 80% of all lung cancers and is responsible for approximately half of all cases of brain metastasis (BM) (4, 5). Compared with NSCLC, small cell lung cancer (SCLC) is associated with less aggressive clinical progression and lower sensitivity to radiochemotherapy, resulting in a median survival of 1-2 months (6, 7). SCLC accounts for approximately 14% of cases, with 10% of patients presenting with BM at initial diagnosis, resulting in limited survival (8, 9). There is a pressing need for enhanced therapeutic approaches to overcome this formidable obstacle.

The primary therapeutic modalities for BM from LCs include whole-brain radiotherapy (WBRT), stereotactic radiotherapy (SRT), chemotherapy, and surgical intervention, which are often used sequentially or in combination. Surgical procedure plays a pivotal role in BM management by alleviating symptoms, allowing the acquisition of pathological samples, and distinguishing between radiation necrosis and tumor regrowth (10, 11). Progress in targeted immunotherapies has enhanced the efficacy of stereotactic radiosurgery, and although WBRT is typically recommended, it remains a crucial salvage therapy for treating BM (12–14). Despite the promising clinical benefits of using antibodies that block programmed death 1/ligand 1 (PD-1/L1) and inhibitors targeting disease-driving tyrosine kinases, the median 5-year overall survival remains less than 5% in BM from LCs (6, 15–17). Given the intricate nature and the ongoing debate regarding the optimal treatment options for this prevalent condition, it is essential to conduct a research on progression and prognosis through existing datasets.

Several models have been introduced to predict BM in LCs on the basis of clinical characteristics (18). Significant factors contribute to prolonged survival of BM from LCs, including age ≤ 65 years, female, fewer BM sites, Karnofsky performance status ≥80, tumor volume ≤10 cm^3^, absence of extracerebral metastases, and a neurologically asymptomatic status (19). Considering the rapid advancements in the management of BM from LCs, these studies have employed relatively limited and potentially biased datasets, while also lacking reliable validation (20, 21). Moreover, the predictive value of these research models has not been adequately explained. Numerous studies have confirmed that BM from LCs is closely associated with the immune status of lymphocytes and monocytes in patients (5, 11, 13). Therefore, if the relationship between the predictive models and the immune levels of patients can be demonstrated, the value of the predictive models will become more compelling.

This study thoroughly delves into the comprehensive, dynamically updated information about cancers from the Surveillance, Epidemiology, and End Results (SEER) database. It specifically focused on the economic status, living conditions, medical features, and unfavorable factors associated with BM from LCs. Additionally, it identified key prognostic factors and developed a tailored predictive model for this scenario, potentially offering valuable insights for both clinical management and healthcare economic policy-making.

## Materials and methods

### Study population and data collection

This study collected patient records on 582649 cases of lung carcinomas from 17 registries within the SEER database via SEER*Stat software version 8.4.3, covering the years 2010-2021. It enrolled participants aged 18 years or older with detailed clinical documentation and a definitive diagnosis of primary LCs. The exclusion criterion was patients whose essential variables, including survival duration and prognosis, were incomplete. Ultimately, 86,763 cases diagnosed with LCs were included. Among them, 15,180 cases were identified as LCs with BM and 71,583 without BM. Then, the BM population was stratified into training and validation cohorts at a 7:3 ratio.

The participants included in the study were profiled according to several key variables, including socioeconomic status (race, marital status, income), demographic characteristics (gender, age), tumor attributes (primary site in the lung, tumor size, histopathology, WHO grade, TNM stage), and treatment protocols (primary site surgery, radiotherapy, chemotherapy). Tumor sizes and time intervals from diagnosis to therapy initiation were stratified according to the median value within each cohort.

### Cox analyses and nomogram construction

Univariable and multivariable Cox regression analyses were conducted to identify independent prognostic factors for overall survival. Variables such as gender, age, marital status, income, primary site, tumor size, histology type, T/N stage, node-positive status, diagnosis to therapy, primary site surgery, radiotherapy, and chemotherapy were incorporated to construct a nomogram model through machine learning. The scores of patients was calculated based on the nomogram model. The computer will calculate the score for each patient based on the model after inputting various variables into the system. The model’s effectiveness and credibility were evaluated using receiver operating characteristics (ROC), with values ranging from 0.5-1.0, where a higher value signifies a more remarkable discriminative ability. Calibration plots and decision curve analysis were employed to assess the concordance between the predicted outcomes and the observed data.

### Model evaluation and interpretation

A range of machine learning approaches were employed through the “tidymodels” R package to assess the overall effectiveness of the model. The importance of variables in the nomogram was evaluated using SHAP values and multiple metrics, including enhancements in the mean accuracy and the Gini coefficient. Moreover, an analysis of SHAP interaction values was conducted to demonstrate the impact of variable interactions on the predicted outcomes.

### Establishment of the research cohort

From October to December 2024, patients with lung cancer and brain metastases (n=20) who sought treatment at the General Hospital of Tibet Military Command were enrolled in this study cohort. The clinical data of these patients were collected and evaluated using our prediction model. Based on the scores obtained from the evaluation, patients were divided into a high grades group (n=12) and a low grades group (n=8). All patients provided signed informed consent. This study was certified and supported by the Ethics Committee of the General Hospital of Tibet Military Command (20241208001).

### Peripheral blood mononuclear cell isolation

Blood samples were collected with EDTA (Ethylene diamine tetraacetic acid) to prevent coagulation. Carefully layer the diluted blood onto the surface of a Ficoll-Hypaque Plus solution. Centrifuge the tube at 400 x g for 30 minutes at room temperature without brake. The middle layer consists of PBMCs was collected into a new sterile conical tube for next analysis.

### FACS analysis

PBMCs were stained with conjugated antibodies as follws: L/D-APC/Cy7, CD45-PerCp (2D1), CD14-FITC (63D3), CD16-APC (3G8), (all from Biolegend). The subsets of monocytes were recognized by both CD14 and CD16.

### Enzyme-linked immunosorbent assay analysis

The plasma cytokines and chemokines, interleukin (IL)–2, IL-6, IL-10 and tumor necrosis factor (TNF)-α were detected using ELISA kit (R&D System). The optical density (OD) value of the experimental results was read on a microplate reader (Bio-Rad).

### Statistical analyses

Data analysis was performed with R software (R Foundation, Vienna, Austria, version 4.1.2). Categorical data were evaluated by the chi-square test or Fisher’s exact test, while differences in Kaplan–Meier survival curves were determined by the log-rank test. Univariable and multivariable Cox regression analyses were conducted to investigate independent prognostic factors among the clinical variables, while analysis of variance (ANOVA) was employed to identify significant differences among multiple groups. If the ANOVA yielded statistically significant findings, *post hoc* comparisons were conducted using Tukey’s honestly significant difference test. The Mann–Whitney U test was employed to compare two independent samples. Dunn’s test was applied for nonparametric comparisons among multiple independent samples with data that did not follow a normal distribution.

Variables with a *p* value < 0.05 in univariate analysis were advanced to multivariate Cox regression analysis. The “forestplot” package in R was used to display the *p* value, hazard ratio, and 95% confidence interval of each variable. The performance of the nomogram in predicting the prognosis of BM patients was evaluated by constructing ROC curves in the “rms” R package. Further model interpretation, including the SHAP summary plot and interaction analysis, was carried out using the “xgboost” and “shapviz” R packages. The “ingredients” package helps determine variable importance, pinpointing the features most crucial to the model’s predictive accuracy. All the statistical tests were two-sided, with a *p* value < 0.05 indicating statistical significance.

## Results

### Clinical characteristics

**Figure 1:**
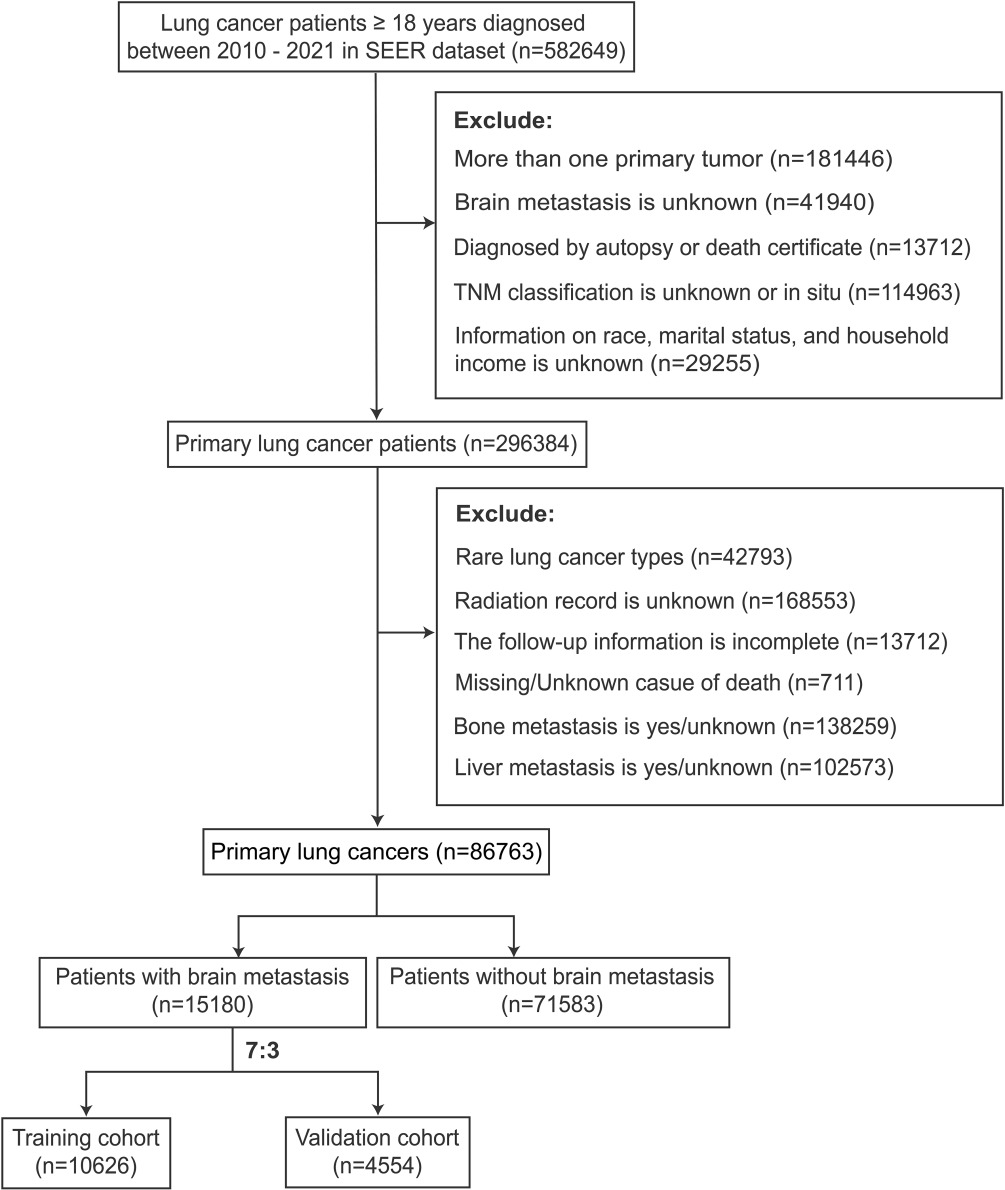
Flowchart depicting the selection criteria for LCs with and without BM.

The participant selection criteria flow diagram is illustrated in **Figure 1**. A summary of the clinical features is shown in **Table 1**. The research findings indicated that BM was present in 17.49% of patients diagnosed with LCs, with the majority of cases occurring in individuals aged ≥ 50 years (93.8%). Among all patients with lung cancer, males constitute 52.4%. Specifically, in the group of patients with lung cancer that has metastasized to the brain, males account for 50.6%. LCs with BM primarily originated from the upper lung lobe (62.5%), characterized by a larger average diameter of 45.0 mm and predominantly adenocarcinoma histology (61.7%). Additionally, significant differences were observed in primary site surgery, radiotherapy, and chemotherapy between the LCs without and with BM cohorts (*p* < 0.001).

**Table 1:** Demographics and clinicopathological features.

Variables	Lung carcinoma (n=86763)	Without BM (n= 71583)	BM (n= 15180)	*p* value
Year at diagnosis, n (%)				<0.001
2010-2015	44136 (50.9)	36084 (50.4)	8052 (53.1)	
2016-2021	42627 (49.1)	35499 (49.6)	7128 (46.9)	
Gender, n (%)				<0.001
Male	45435 (52.4)	37754 (52.7)	7681 (50.6)	
Female	41328 (47.6)	33829 (47.3)	7499 (49.4)	
Age, n (%) years				<0.001
<40	365 (0.4)	250 (0.4)	115 (0.8)	
40-49	2849 (3.3)	2023 (2.8)	826 (5.4)	
50-59	15361 (17.7)	11469 (16.0)	3892 (25.6)	
60-69	28703 (33.1)	23099 (32.3)	5604 (36.9)	
70-79	26881 (31.0)	23189 (32.4)	3692 (24.3)	
>80	12604 (14.5)	11553 (16.1)	1051 (6.9)	
Race, n (%)				<0.001
American Indian	569 (0.7)	479 (0.7)	90 (0.6)	
Asian or Pacific Islander	5332 (6.2)	4105 (5.7)	1227 (8.1)	
Black	10718 (12.4)	8700 (12.2)	2018 (13.3)	
White	70144 (80.9)	58299 (81.4)	11845 (78.0)	
Marital status, n (%)				<0.001
Divorced	12475 (14.4)	10345 (14.5)	2130 (14.0)	
Married	44803 (51.6)	36687 (51.3)	8116 (53.5)	
Unmarried	14630 (16.7)	11549 (16.1)	3081 (20.3)	
Widowed	14855 (17.1)	13002 (18.2)	1853 (12.2)	
Incomes, n (%) k				<0.001
<40	2721 (3.1)	2287 (3.2)	434 (2.9)	
40 - 50	8568 (9.9)	7163 (10.1)	1405 (9.3)	
50 - 60	11914 (13.7)	9884 (13.8)	2030 (13.4)	
60 - 70	16006 (18.5)	13363 (18.7)	2643 (17.4)	
70 - 80	16487 (19.0)	13534 (18.9)	2953 (19.5)	
80 - 90	10905 (12.6)	8990 (12.6)	1915 (12.6)	
90 - 100	8629 (10.0)	7036 (9.8)	1593 (10.5)	
>100	11533 (13.3)	9326 (13.0)	2207 (14.5)	
Primary site, n (%)				<0.001
Lower lobe	23194 (26.7)	19249 (26.9)	3945 (26.0)	
Main bronchus	5297 (6.1)	4442 (6.2)	855 (5.6)	
Middle lobe	3799 (4.4)	3063 (4.3)	736 (4.9)	
Overlapping lesion	883 (1.0)	722 (1.0)	161 (1.1)	
Upper lobe	53590 (61.8)	44107 (61.6)	9483 (62.5)	
Tumor size (mm)	41.0 (25.0, 63.0)	40.0 (25.0, 62.0)	45.0 (30.0, 66.0)	<0.001
Differentiated degree, n (%)				<0.001
Moderate	3996 (4.6)	3629 (51.0)	367 (5.4)	
Poor	5398 (6.2)	4425 (6.2)	973 (6.4)	
Undifferentiated	396 (0.5)	312 (0.4)	84 (0.6)	
Well	872 (1.0)	834 (1.2)	38 (0.3)	
Unknown	76101 (87.7)	62383 (87.2)	13718 (90.4)	
Grade stage, n (%)				<0.001
I	1734 (2.0)	1569 (2.2)	165 (1.1)	
II	8933 (10.3)	7925 (11.1)	1008 (6.6)	
III	16510 (19.0)	13585 (19.0)	2925 (19.3)	
IV	1818 (2.1)	1461 (2.0)	357 (2.4)	
Unknown	57768 (66.6)	47043 (65.7)	10725 (70.7)	
T stage, n (%)				<0.001
T1	56700 (65.4)	47217 (66.0)	9483 (62.5)	
T2	23183 (26.7)	18892 (26.4)	4291 (28.3)	
T3	3206 (3.7)	2600 (3.6)	606 (4.0)	
T4	3674 (4.2)	2874 (4.0)	800 (5.3)	
N stage, n (%)				<0.001
N0	30464 (35.1)	47217 (66.0)	3749 (37.3)	
N1	7636 (8.8)	18892 (26.4)	1443 (8.7)	
N2	35484 (40.9)	2600 (3.6)	6918 (40.0)	
N3	13179 (15.2)	2874 (4.0)	3070 (14.1)	
M stage, n (%)				<0.001
M0	59371 (68.4)	59371 (83.0)	0 (0)	
M1	27392 (31.6)	12212 (17.1)	15180 (100.0)	
Node positive, n (%)				<0.001
No	59314 (68.4)	47564 (66.4)	11750 (77.4)	
Yes	27449 (31.6)	24019 (33.6)	3430 (22.6)	
Histology type, n (%)				<0.001
Adenocarcinoma	38013 (43.8)	28653 (40.0)	9360 (61.7)	
Squamous cell carcinoma	29312 (33.8)	27557 (38.5)	1755 (11.6)	
Large cell carcinoma	1293 (1.5)	950 (1.3)	343 (2.3)	
Non-small cell carcinoma	5040 (5.8)	3955 (5.5)	1085 (7.2)	
Squamous cell carcinoma	29312 (33.8)	27557 (38.5)	1755 (11.6)	
Diagnose to therapy (days)	31.0 (11.0, 55.0)	35.0 (15.0, 59.0)	13.0 (4.0, 29.0)	<0.001
Primary site surgery, n (%)				<0.001
No	80810 (93.1)	66106 (92.4)	14704 (96.9)	
Yes	5953 (6.9)	5477 (7.7)	476 (3.19)	
Radiotherapy, n (%)				<0.001
No	3127 (3.6)	2784 (3.9)	343 (2.3)	
Yes	83636 (96.4)	68799 (96.1)	14837 (97.7)	
Chemotherapy, n (%)				0.051
No/Unknown	31977 (36.9)	26488 (37.0)	5489 (36.2)	
Yes	54786 (63.1)	45095 (63.0)	9691 (63.8)	
Cancer cause death, n (%)				<0.001
No	32186 (37.1)	29365 (41.0)	2821 (18.6)	
Yes	54577 (62.9)	42218 (59.0)	12359 (81.4)	
Others cause death, n (%)				<0.001
No	76966 (88.7)	62366 (87.1)	14600 (96.2)	
Yes	9797 (11.3)	9217 (12.9)	580 (3.8)	
Status on OS, n (%)				<0.001
Alive	22389 (25.8)	20148 (28.2)	2241 (14.8)	
Dead	64374 (74.2)	51435 (71.9)	12939 (85.2)	

1k = 1,000 USD.

### Survival time

Patients with BM presented an overall worse prognosis, with a median survival of 8 months, compared with 16 months for their counterparts (*p <*0.001). BM originating from LCs has been shown to decrease survival time in older individuals, males, widowed individuals, and American Indian individuals (*p <*0.001). Higher economic status correlated with prolonged survival duration, possibly due to increased access to expensive treatment options (*p <*0.001). Additionally, shorter intervals between LC diagnosis and treatment initiation, smaller tumor volumes, lower T/N stages, the absence of positive lymph nodes, good differentiation, and lower-grade staging were associated with better survival, particularly in patients with adenocarcinoma pathology (*p <*0.001). Furthermore, undergoing primary tumor surgery, radiotherapy, or chemotherapy has been associated with prolonged survival, even with significant effects observed from both surgery and chemotherapy (*p <*0.001) (**Supplementary Table 1**). The survival time of the subgroups within the BM cohort was further delineated, as presented in **Supplementary Table 1**.

### Univariate and multivariate Cox regression analyses

Various variables, such as gender, age, marital status, income, primary site of LCs, tumor size, histology type, T/N stage, lymph node status, and interval from diagnosis to therapy, with *p* ≤ 0.05, were identified as potentially significant and selected for multivariate analysis. Multivariate Cox regression independently validated these variables as prognostic indicators of overall survival, underscoring their crucial role in predicting the prognosis of patients with BM (**Supplementary Table 2**).

**Figure 2:**
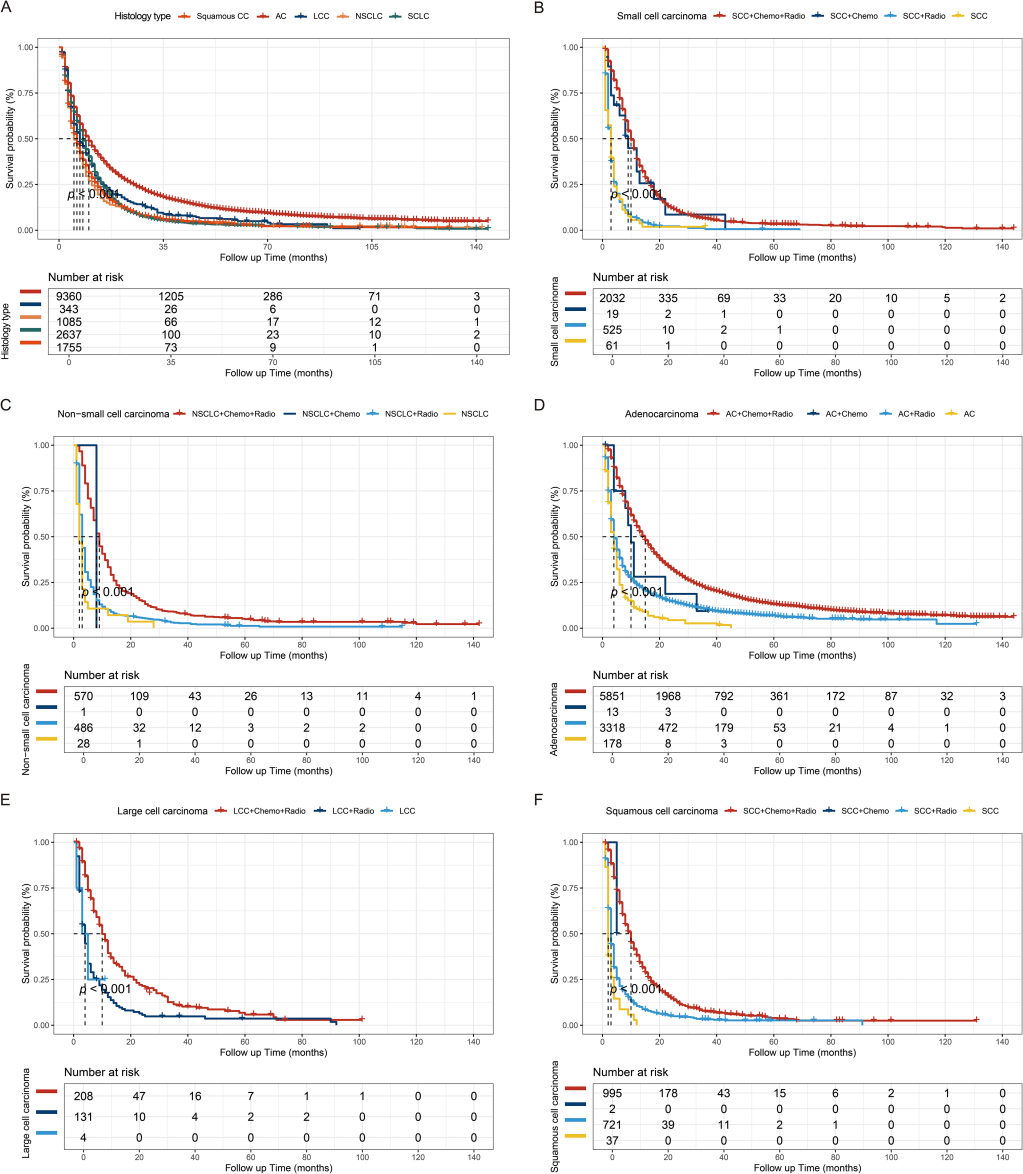
Kaplan-Meier survival curves. **(A)** Kaplan-Meier survival curves stratified by histological subtype (*p* < 0.001). The treatment regimens for small cell carcinoma **(B)**, non-small cell carcinoma **(C)**, adenocarcinoma **(D)**, large cell carcinoma **(E)**, and squamous cell carcinoma **(F)**. AC, Adenocarcinoma; LCC, Large cell carcinoma; NSCLC, Non-small cell carcinoma; SCLC, Small cell carcinoma; SCC, squamous cell carcinoma.

Further examination through Kaplan-Meier analysis assessed the impact of histological type and radiochemotherapy on overall survival. BM with adenocarcinoma histology was associated with favorable outcomes in the overall analysis. Moreover, irrespective of the specific pathological subtype, adjuvant radiochemotherapy was associated with improved prognosis. These findings highlight the effectiveness of combined therapy in enhancing outcomes for BM originating from LCs (22). Additionally, with the exception of large cell carcinoma, the efficacy of chemotherapy alone appears to surpass that of radiotherapy alone, among other specific histological subtypes (**Figure 2**).

### Construction and evaluation of the nomogram

**Figure 3:**
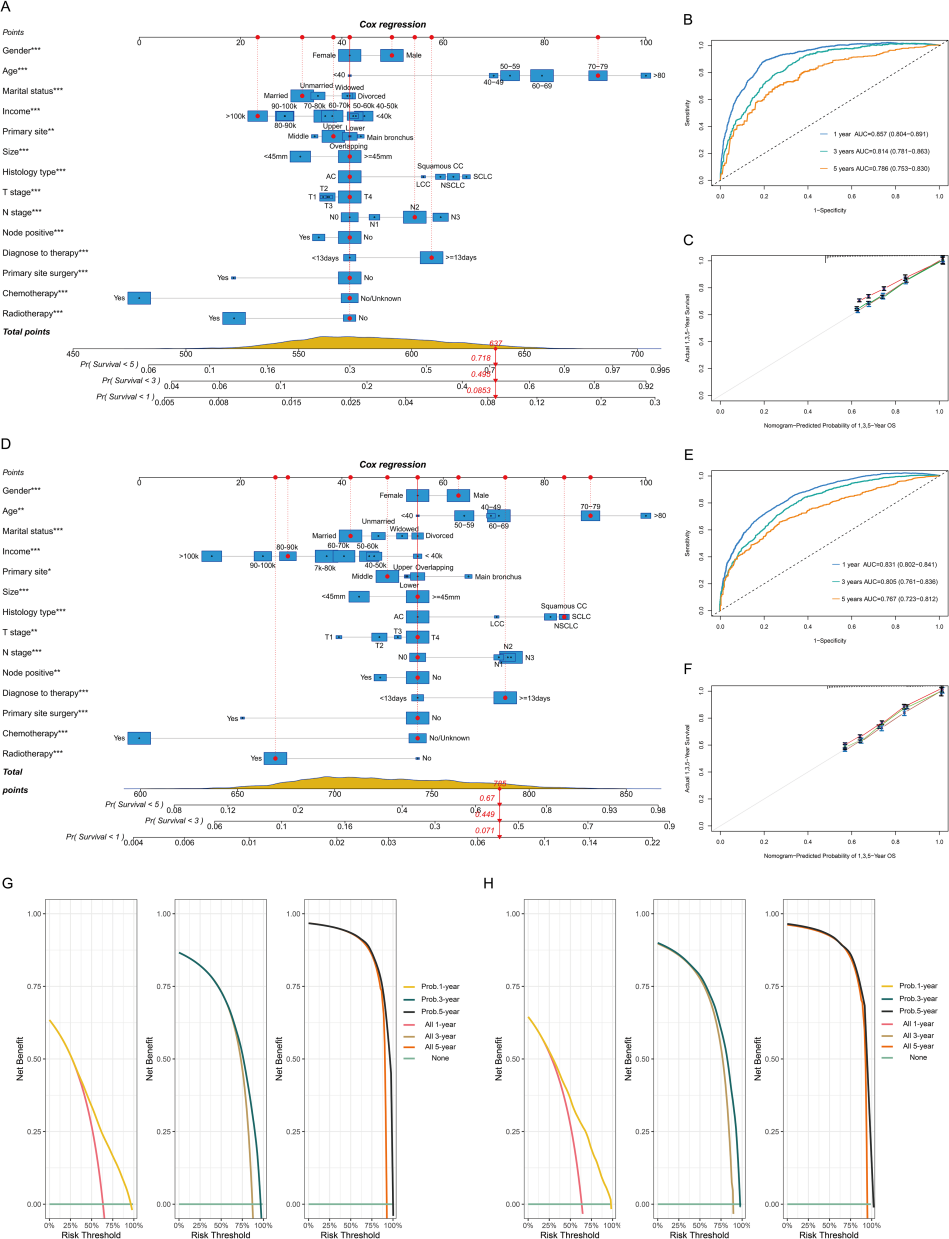
Model construction and validation for 1-, 3-, and 5-year overall survival (OS). Nomogram model, receiver operating characteristic curve, and calibration plots for OS in the BM training cohort **(A-C)** and validation cohort **(D-F)**. Decision curve for the prognostic model in the BM training **(G)** and validation cohorts **(H)**. 1k=1,000 USD.

A nomogram was developed to forecast the probabilities of overall survival at 1, 3, and 5 years on the basis of 14 key variables in the BM training cohort, as depicted in **Figure 3A**. The nomogram demonstrated strong predictive performance, with AUC values of 0.857 (95% CI 0.804-0.891) for one year, 0.814 (95% CI 0.781-0.863) for three years, and 0.786 (95% CI 0.753-0.830) for five years (**Figure 3B**). The calibration curve and decision curve analysis for the 1-, 3-, and 5-year survival probabilities revealed strong performance between the predicted and observed outcomes (**Figures 3C, G**). The model was internally validated, showing strong predictive capability (**Figures 3D–F, H**).

### Validation of the performance of the prediction model

**Figure 4:**
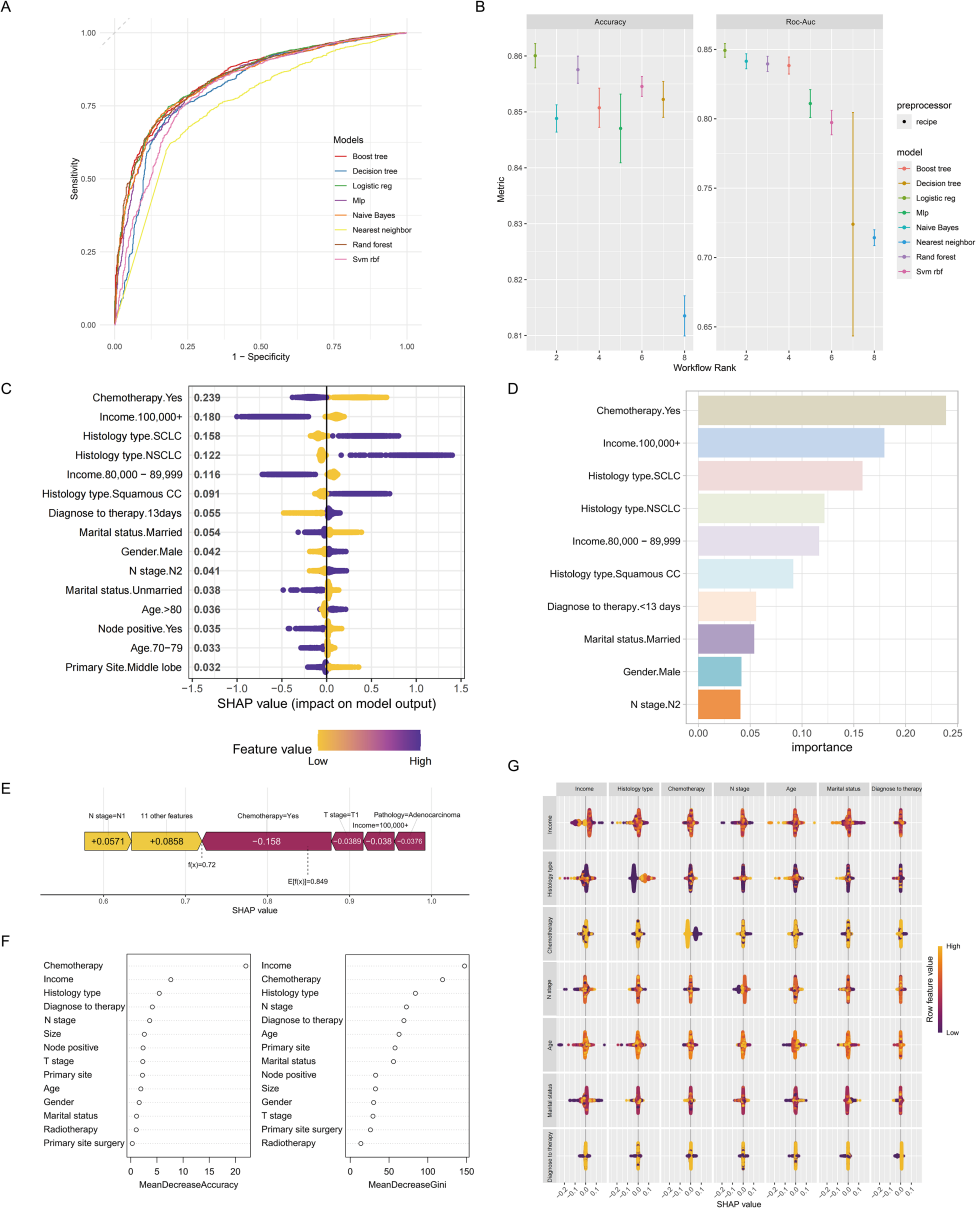
Machine learning approaches for the predictive model. Time-dependent receiver operating characteristic curve **(A)** and accuracy **(B)** of the machine learning methods used for the predictive model. **(C)** The SHAP summary plot revealed a diverse distribution of points, indicating that chemotherapy, income, and histology type substantially impacted the model’s performance. **(D)** A Bar graph depicting the top ten variables that exhibited the most significant impact on the predictive accuracy of the model. **(E)** The SHAP value of each variable in the 306th patient. **(F)** Mean decrease in accuracy (panel left) and mean decrease in the Gini coefficient (panel right). They also emphasized the notable influence of chemotherapy, income, and histology type on the model’s predictive accuracy. **(G)** SHAP interactions among variables.

Various machine learning methods were employed to validate the overall performance of the model, demonstrating a relatively strong power. Specifically, logistic regression displayed the most favorable predictive capability, with an AUC value of 0.844 (95% CI 0.764-0.871) and an accuracy of approximately 0.860 (95% CI 0.803-0.921). Conversely, the nearest neighbor method exhibited the lowest predictive performance and accuracy (**Figures 4A, B**). Moreover, a total of 14 key variables were identified as significantly contributing to the predictive effectiveness based on their high mean absolute SHAP scores. The SHAP summary plot displayed a varied distribution of points, highlighting the substantial impacts of chemotherapy, income condition, histology type, and interval between diagnosis and therapy on the model performance (**Figures 4C, D**). The SHAP value of the model prediction for the 306th patient was 0.849, along with significant contributions from important variables, particularly the weight assigned to receiving chemotherapy being the highest (**Figure 4E**). A decrease in the mean value of accuracy and the Gini coefficient suggests a minimal impact on the model’s performance, while an increase indicates a significant improvement. These findings underscore the importance of chemotherapy, income condition, histology type, and the interval between diagnosis and therapy in enhancing the predictive performance of the model (**Figure 4F**). Additionally, SHAP interaction analysis demonstrated that income has a notable interaction effect with other variables (**Figure 4G**).

### The association between the predictive model and patient’s immune status

**Figure 5:**
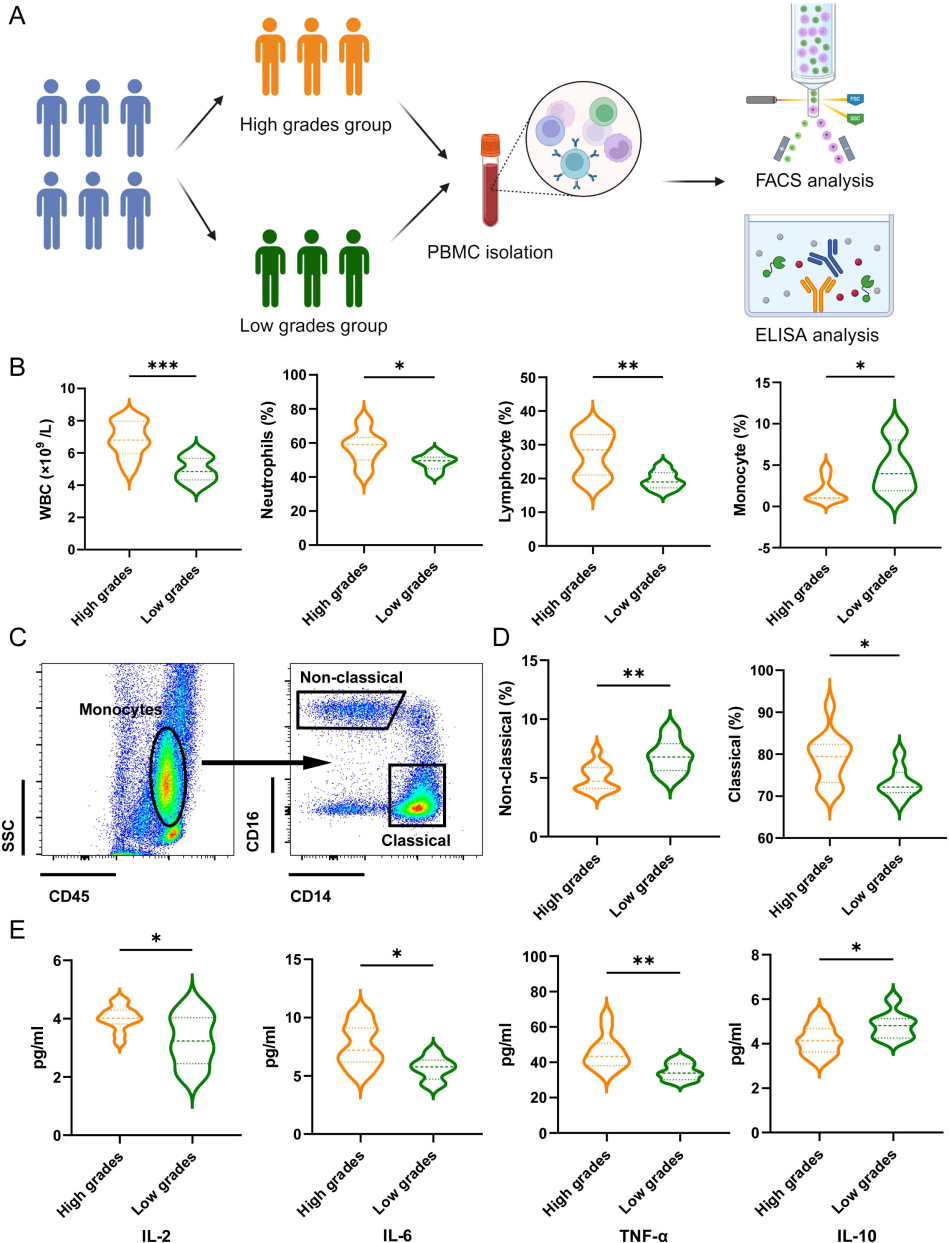
The association between the predictive model and patient’s inflammatory response. **(A)** Schematic illustration showing patients grouping, PBMC isolation, FACS analysis and ELISA analysis. **(B)** Effects of the grades of predictive model on WBC counts, proportion of neutrophils, lymphocyte and monocytes. Data are presented with the Violin Diagram. *P < 0.05, **P < 0.01, ***P < 0.001. **(C)** Representative FACS plots of the distributions of monocyte and the subsets in patients of LCs with BM. **(D)** Effects of the grades of predictive model on the proportion of non-classical and classical monocytes. Data are presented with the Violin Diagram. *P < 0.05, **P < 0.01. **(E)** Effects of the grades of predictive model on the level of IL-2, IL-6, TNF-α and IL-10. Data are presented with the Violin Diagram. *P < 0.05, **P < 0.01.

Next, we validated the relationship between the predictive model and the immunological status of patients through a clinical cohort. We collected data from 20 lung cancer patients with brain metastases who were treated at the Tibet Military Region General Hospital. Based on the predictive model scores, patients were divided into a high grades group (n=12) and a low grades group (n=8). We gathered peripheral blood mononuclear cells (PBMCs) and plasma from these patients for flow cytometry and ELISA analyses (**Figure 5A**). We compared the routine blood test results between the two groups. Compared to the low grades group, the high grades group exhibited significantly elevated white blood cell (WBC) counts, neutrophil proportions, and lymphocyte proportions, while the monocyte proportion decreased (**Figure 5B**). Further monocyte subset analysis indicated that the high grades group primarily showed a deficiency in atypical monocyte subsets (**Figures 5C, D**). ELISA results revealed that levels of IL-2, IL-6, and TNF-α were significantly higher in the high grades group, whereas IL-10 levels were reduced (**Figure 5E**).

## Discussion

BM, characterized by its distinct cell types, compromised blood-brain barrier, metabolic dysregulation, and specific immune milieu, exhibits aggressive biological behavior, impacting the metastatic cascade and therapeutic responses (23). Moreover, a confluence of socioeconomic condition, pathological characteristics, and treatment regimens affect the prognosis of BM patients (14, 24, 25). Owing to the significant prevalence and mortality of BM from LCs, identifying prognostic factors to improve overall survival is imperative.

These findings of the study highlighted the notable disparities in the prognosis of BM across demographic subgroups, including age, ethnicity, and socioeconomic status. Epidemiological studies have investigated the relationship between gender and the prevalence of BM in individuals with LCs. Males exhibited a higher prevalence and more adverse outcomes than females did, indicating the presence of underlying biological variations that may have contributed to this discrepancy (26, 27). Age is a notable prognostic indicator for patients with BM, as both the incidence and mortality of BM tend to rise with increasing age, which is particularly prominent in individuals aged 70 years and older (28). Socioeconomic status has the potential to affect overall survival by impacting patients’ mental health, treatment compliance, and access to medical care (29). This emphasized the importance of exercising caution when generalizing study results to diverse population subgroups, aligning with previous reports (30, 31).

Histopathological features, including tumor size, lymph node positivity, T/N stage, and pathology type, all significantly affect BM prognosis. Larger tumors are associated with an increased risk of metastasis and pose challenges for achieving complete surgical resection, whether at the primary site or in the brain (32, 33). Our study also revealed that N stage and lymph node positivity are independent prognostic factors for individuals with BM. In LCs, lymph node metastasis can be categorized into intrathoracic and extrathoracic spread. Extrathoracic lymph node metastasis eliminates the opportunity for surgical intervention, significantly reducing both the survival time and quality of life (34). Additionally, we confirmed a distinct association between the advancement of T or N stage and tumor growth, resulting in a gradual decrease in survival. Meanwhile, patients with brain metastases from SCLC had the poorest prognosis (median 5 months), making it a significant prognostic factor (HR: 1.56, 95% CI: 1.46-1.66). Consistent with prior researches (35–37), these findings provide further validation of the strong correlation between these factors and the clinical prognosis of BM from LCs.

The approach to treating BM from LCs involves a combination of local interventions, such as radiation and surgery, as well as systemic therapies (38–40). WBRT was historically considered the primary treatment modality for addressing gross and minor lung or intracranial lesions, reducing the risk of local and distant intracranial recurrence. However, advances in systemic therapy and radiotherapy delivery have led to a diminished role for WBRT (41, 42). Although chemotherapeutic agents traditionally have limited activity in the brain due to the blood–brain barrier, most patients with BM have concurrent extrathoracic disease that requires systemic therapy (40). The efficacy of various treatment modalities exhibits substantial variability in each pathology subtype of BM in LCs. Although combined chemoradiotherapy generally yields the most favorable outcomes across these subtypes, their effects differ markedly. For instance, in small cell lung carcinoma brain metastases, radiotherapy alone does not significantly enhance survival, and combined chemoradiotherapy does not offer an advantage over chemotherapy alone. This finding contrasts with previous results that small cell lung carcinoma is responsive to radiotherapy and can derive substantial benefits from such radiotherapy. Specifically, the sensitivity of small cell lung carcinoma to radiotherapy is closely associated with tumor differentiation and TNM staging (43, 44). Conversely, other pathological subtypes demonstrate notable improvements in prognosis with either radiotherapy alone or in combination with chemotherapy. Regrettably, only 3.19% of individuals with BM underwent surgery for the primary lung lesion, and 63.8% received chemotherapy in this study. Additionally, information about intracranial BM size, number, and surgical protocol needs to be clarified, introducing potential bias in the interpretation of the results.

The current study identified 14 independent indicators associated with the prognosis of patients with lung cancer BM, such as socioeconomic status, demographic characteristics, tumor biological features, and treatment protocols. By integrating these variables, a nomogram was constructed, showing superior accuracy in predicting outcomes, as evidenced by the AUC values of 0.857 (1 year), 0.814 (3 years), and 0.786 (5 years). The predictive model stands out from those of previous studies by incorporating a diverse array of variables pertinent to the prognosis of BM from LCs, setting training and validation cohorts, and employing multiple machine learning approaches to assess model efficacy, thereby bolstering its credibility (45–47).

The hematological profile comparison revealed that the high grades group exhibited a significant elevation in WBC counts, neutrophil proportions, and lymphocyte proportions relative to the low grades group. These results suggest a distinct immunological landscape in patients with higher predictive grades, potentially indicative of an activated immune response or altered immune cell distribution. Further monocyte subset analysis identified a notable deficiency in atypical monocyte subsets within the high grades group. This finding underscores the complexity of immune modulation in these patients and hints at potential dysregulation in monocyte lineage differentiation or function (5, 12). The cytokine profiles are consistent with a pro-inflammatory state, suggesting that higher predictive scores may be associated with heightened immune activation or inflammation. Taken together, these results highlight the potential utility of the predictive model in identifying patients with distinct immunological characteristics (25, 40). The model’s ability to stratify patients based on immunological markers could pave the way for personalized therapeutic strategies, particularly in the context of immunotherapy.

This retrospective study is constrained by its reliance on a particular cohort from public databases. The exclusion of cases with incomplete data may introduce selection bias, and there may be inconsistency in the definition of variables across data sources. BM frequently occurs in the cerebral hemispheres, cerebellum, and brainstem, while leptomeningeal metastases, although less common, have an inferior prognosis (48). The lack of imaging specificity regarding the quantity, volume, and extent of BM represents a notable omission with potential implications for the predictive outcomes of patients (49). The SEER database does not present molecular markers, including EGFR, ALK, and ROS1, which are critical in guiding the prognosis and therapy for lung cancer BM. Despite these limitations, the findings of this study are scientifically sound and provide valuable guidance for the clinical management of BM originating from LCs.

## Conclusions

BM originating from LCs presents a complex and challenging clinical scenario influenced by diverse economic, social, and medical factors. This study pinpointed the primary risk factors affecting progression and prognosis, constructing a nomogram model for this condition. The predictive model could serve as a valuable tool for both clinical management and healthcare provider decision-making.

## Data Availability

The original contributions presented in the study are included in the article/**Supplementary Material**. Further inquiries can be directed to the corresponding author.
