# Supplementary material for: Developing a predictive model and uncovering immune influences on prognosis for brain metastasis from lung carcinomas
Source: Front Oncol. 2025 Mar 3;15:1554242. doi: 10.3389/fonc.2025.1554242 (PMC11911169; doi:10.3389/fonc.2025.1554242)
Supplement: Supplementary file 1 [file Table1.docx]

Supplementary table 1 Survival time of lung carcinoma without and with brain metastasis

| Variables (Months) | Lung carcinoma  (n=86763) | Without BM  (n= 71583) | BM  (n= 15180) | *p* value |
| --- | --- | --- | --- | --- |
| Survival time | 15 (7, 31) | 16 (8, 34) | 8 (4, 17) | < 0.001 |
| Year at diagnosis ^§^ |  |  |  |  |
| 2010-2015 | 15 (7, 34) | 17 (8, 39) | 7 (4, 15) | < 0.001 |
| 2016-2021 | 14 (7, 29) | 16 (8, 31) | 8 (4, 19) | < 0.001 |
| Gender ^§^ |  |  |  |  |
| Male | 13 (6, 28) | 15 (7, 31) | 7 (3, 15) | < 0.001 |
| Female | 16 (7, 34) | 18 (9, 37) | 9 (4, 19) | < 0.001 |
| Age ^＆^ |  |  |  |  |
| <40 years | 19 (9, 41) | 18 (9, 41) | 20 (9, 40.5) | 0.995 |
| 40 - 49 years | 16 (8, 35) | 19 (10, 42) | 11 (6, 23) | < 0.001 |
| 50 - 59 years | 15 (8, 33) | 18 (9, 38) | 10 (5, 20) | < 0.001 |
| 60 - 69 years | 15 (7, 31) | 17 (8, 35) | 8 (4, 18) | < 0.001 |
| 70 - 79 years | 14 (6, 30) | 16 (8, 32) | 6 (3, 13) | < 0.001 |
| >80 years | 14 (6, 29) | 15 (7, 31) | 4 (2, 9) | < 0.001 |
| Race ^£^ |  |  |  |  |
| American Indian | 14 (7, 29) | 16 (8, 31) | 7 (3, 14) | < 0.001 |
| Asian or Pacific Islander | 17 (7, 36) | 19 (8, 39) | 12 (5, 25) | < 0.001 |
| Black | 14 (7, 30) | 16 (8, 34) | 8 (4, 16) | < 0.001 |
| White | 14 (7, 31) | 16 (8, 34) | 8 (3, 16) | < 0.001 |
| Marital status ^※^ |  |  |  |  |
| Divorced | 14 (7, 30) | 16 (8, 33) | 7 (3, 15) | < 0.001 |
| Married | 15 (7, 32) | 17 (8, 35) | 9 (4, 19) | < 0.001 |
| Unmarried | 13 (6, 29) | 15 (8, 32) | 8 (4, 16) | < 0.001 |
| Widowed | 14 (6, 30) | 16 (7, 33) | 6 (3, 13) | < 0.001 |
| Incomes ^#^ |  |  |  |  |
| <40k | 13 (6, 28) | 14 (7, 31) | 7 (3, 13) | < 0.001 |
| 40 - 50k | 13 (6, 28) | 15 (7, 31) | 6 (3, 14) | < 0.001 |
| 50 - 60k | 14 (6, 29) | 15 (8, 32) | 7 (3, 14) | < 0.001 |
| 60 - 70k | 14 (7, 31) | 16 (8, 34) | 7 (3, 15) | < 0.001 |
| 70 - 80k | 15 (7, 32.5) | 17 (8, 36) | 8 (4, 17) | < 0.001 |
| 80 - 90k | 15 (7, 31) | 17 (8, 33) | 8 (4, 18) | < 0.001 |
| 90 - 100k | 15 (7, 31) | 16 (8, 34) | 8 (4, 18) | < 0.001 |
| >100k | 16 (7, 33) | 18 (8, 36) | 10 (4, 23) | < 0.001 |
| Primary site ^¶^ |  |  |  |  |
| Lower lobe | 14 (7, 30) | 16 (8, 33) | 8 (3, 16) | < 0.001 |
| Main bronchus | 11 (5, 23) | 12 (6, 25) | 6 (3, 13.5) | < 0.001 |
| Middle lobe | 16 (7, 32.5) | 17 (8, 35) | 9 (4, 20) | < 0.001 |
| Overlapping lesion | 11 (5, 24) | 12 (6, 27) | 8 (3, 14) | < 0.001 |
| Upper lobe | 15 (7, 32) | 17 (8, 35) | 8 (4, 17) | < 0.001 |
| Tumor size ^§^ |  |  |  |  |
| >=median size | 12 (6, 24) | 13 (6, 27) | 7 (3, 15) | < 0.001 |
| <median size | 18 (8, 37) | 20 (10, 40) | 9 (4, 19) | < 0.001 |
| Differentiated degree |  |  |  |  |
| Moderate | 14 (7, 25) | 14 (7, 26) | 10 (4, 18) | < 0.001 |
| Poor | 12 (6, 23) | 13 (6, 24) | 7 (3, 16) | < 0.001 |
| Undifferentiated | 11 (6, 18) | 12 (7, 19) | 11 (4, 17) | < 0.001 |
| Well | 18 (9, 30) | 18 (9, 30) | 16 (5, 24.8) | < 0.001 |
| Unknown | 15 (7, 32) | 17 (8, 36) | 8 (4, 17) | < 0.001 |
| Grade stage |  |  |  |  |
| I | 28 (12, 56.8) | 31 (14, 59) | 10 (5, 20) | < 0.001 |
| II | 19 (8, 43) | 20 (9, 47) | 9 (4, 21) | < 0.001 |
| III | 15 (7, 35) | 17 (8, 41) | 8 (3, 15) | < 0.001 |
| IV | 14 (7, 30) | 16 (9, 36) | 7 (3, 14) | < 0.001 |
| Unknown | 14 (7, 28) | 16 (8, 31) | 8 (4, 17) | < 0.001 |
| T stage |  |  |  |  |
| T1 | 16 (8, 33) | 18 (9, 36) | 9 (4, 19) | < 0.001 |
| T2 | 15 (7, 37） | 17 (8, 42) | 8 (4, 19) | < 0.001 |
| T3 | 12 (5, 31) | 14 (6, 35) | 8 (3, 19) | < 0.001 |
| T4 | 14 (7, 30) | 16 (8, 33) | 7 (3, 16) | < 0.001 |
| N stage ^¢^ |  |  |  |  |
| N0 | 19 (9, 38) | 21 (10, 40) | 9 (4, 20) | < 0.001 |
| N1 | 15 (7, 30.2) | 17 (8, 33) | 9 (4, 19) | < 0.001 |
| N2 | 13 (6, 28) | 15 (7, 31) | 7 (3, 16) | < 0.001 |
| N3 | 11 (5, 23) | 12 (6, 25) | 7 (3, 15) | < 0.001 |
| M1 stage | 9 (4, 18) | 10 (5, 20) | 8 (4, 17) | < 0.001 |
| Node positive ^§^ |  |  |  |  |
| No | 13 (6, 29) | 15 (7, 32) | 7 (3, 16) | < 0.001 |
| Yes | 17 (8, 36) | 19 (9, 39) | 9 (4, 19) | < 0.001 |
| Histology type ^¶¶^ |  |  |  |  |
| Adenocarcinoma | 16 (7, 35) | 20 (9, 40) | 9 (4, 20) | < 0.001 |
| Squamous cell carcinoma | 14 (7, 29) | 15 (7, 30) | 7 (3, 12) | < 0.001 |
| Large cell carcinoma | 12 (6, 27) | 15 (8, 31.8) | 8 (3, 13.5) | < 0.001 |
| Non-small cell carcinoma | 12 (5, 31) | 15 (7, 39) | 6 (3, 14) | < 0.001 |
| Small cell carcinoma | 13 (7, 24) | 15 (8, 28) | 5 (3, 11) | < 0.001 |
| Diagnose to therapy ^§^ |  |  |  |  |
| ≥ median days | 12 (5, 26) | 14 (7, 30) | 7 (3, 16) | < 0.001 |
| < median days | 18 (9, 35) | 19 (9, 37) | 9 (5, 20) | < 0.001 |
| Primary site surgery ^§^ |  |  |  |  |
| No | 14 (7, 29) | 16 (8, 32) | 8 (3, 16) | < 0.001 |
| Yes | 28 (14, 56) | 30 (14, 57) | 17 (8, 37) | < 0.001 |
| Radiotherapy ^§^ |  |  |  |  |
| No | 7 (3, 16) | 8 (3, 17) | 3 (2, 6) | < 0.001 |
| Yes | 15 (7, 32) | 17 (8, 35) | 8 (4, 17) | < 0.001 |
| Chemotherapy ^§^ |  |  |  |  |
| No/Unknown | 12 (4, 30) | 15 (6, 33) | 4 (2, 8) | < 0.001 |
| Yes | 16 (8, 32) | 17 (9, 34) | 11 (6, 21) | < 0.001 |
| Cancer cause death ^§^ |  |  |  |  |
| No | 26 (10, 52) | 27 (11, 53) | 17 (6, 40) | < 0.001 |
| Yes | 11 (6, 22) | 13 (7, 24) | 7 (3, 14) | < 0.001 |
| Others causes death ^§^ |  |  |  |  |
| No | 14 (7, 29) | 16 (8, 32) | 8 (4, 17) | < 0.001 |
| Yes | 21 (9, 42) | 22 (10, 44) | 7 (3, 18) | < 0.001 |
| Status on OS ^§^ |  |  |  |  |
| Alive | 29 (11, 56) | 29 (11, 58) | 21 (8, 45) | < 0.001 |
| Dead | 12 (6, 24) | 14 (7, 26) | 7 (3, 14) | < 0.001 |

§ The subgroups of the BM populations, including year at diagnosis, gender, size, node-positive, diagnosis to therapy, primary site surgery, radiotherapy, chemotherapy, cancer cause death, other causes death, and status on overall survival (OS), all exhibited statistically significant differences in OS, with *p* < 0.05.

＆ Statistically significant differences in survival time were observed among subgroups according to age (*p* < p.adjust).

£ In the subgroup analysis of race, there were no significant differences in survival time between American Indian and black individuals (*p* = 0.986), American Indian and white individuals (*p* = 0.971), or white and black individuals (*p* = 0.978), but differences were found in other subgroups (*p* < p.adjust).

※ In the subgroup analysis of marital status, there was no significant difference in survival time between the divorced and unmarried variables (*p* = 0.531), but differences were found in the other subgroups (*p* < p.adjust).

# In the income subgroup analysis, the >100k subgroup exhibited statistically significant differences in survival compared with the other subgroups (*p* < p.adjust). 1k=1000USD.

¶ In the primary site subgroup analysis, survival significantly differed between the middle lobe subgroup and the other subgroups (*p* < p.adjust).

¢ In the N stage subgroup analysis, the N0 stage exhibitd statistically significant differences in survival compared to N1, N2, and N3 subgroups (*p* < p.adjust).

¶¶ In the histology subgroup analysis, adenocarcinoma patients exhibited statistically significant differences in survival compared with the other subgroups (*p* < p.adjust).

Supplementary table 2 Univariate and multivariate Cox regression analyses

| Variables | Univariate | | |  | Multivariate | | |
| --- | --- | --- | --- | --- | --- | --- | --- |
|  | HR | 95% CI | *p* value |  | HR | 95% CI | *p* value |
| Gender Male | 1.22 | 1.17-1.27 | < 0.001 |  | 1.19 | 1.14-1.24 | < 0.001 |
| Age <40 years (reference) |  |  |  |  |  |  |  |
| 40 - 49 years | 1.85 | 1.39-2.46 | < 0.001 |  | 1.74 | 1.31-2.32 | 0.001 |
| 50 - 59 years | 2.16 | 1.66-2.85 | < 0.001 |  | 1.91 | 1.45-2.50 | < 0.001 |
| 60 - 69 years | 2.44 | 1.86-3.21 | < 0.001 |  | 2.15 | 1.64-2.82 | < 0.001 |
| 70 - 79 years | 3.20 | 2.44-4.20 | < 0.001 |  | 2.69 | 2.04-3.54 | < 0.001 |
| >80 years | 4.22 | 3.19-5.59 | < 0.001 |  | 3.23 | 2.43-4.29 | < 0.001 |
| Marital status Divorced (reference) |  |  |  |  |  |  |  |
| Married | 0.84 | 0.79-0.90 | < 0.001 |  | 0.84 | 0.79-0.90 | < 0.001 |
| Unmarried | 0.92 | 0.86-0.99 | 0.038 |  | 0.91 | 0.85-0.98 | 0.012 |
| Widowed | 1.17 | 1.09-1.28 | < 0.001 |  |  |  |  |
| Income < 40k (reference) |  |  |  |  |  |  |  |
| 40 - 50k | 0.67 | 0.59-0.76 | 0.617 |  |  |  |  |
| 50 - 60k | 1.03 | 0.91-1.18 | 0.801 |  |  |  |  |
| 60 - 70k | 1.02 | 0.89-1.16 | 0.437 |  |  |  |  |
| 70 - 80k | 0.95 | 0.84-1.08 | 0.033 |  |  |  |  |
| 80 - 90k | 0.74 | 0.65-0.85 | < 0.001 |  | 0.80 | 0.70-0.91 | < 0.001 |
| 90 - 100k | 0.77 | 0.68-0.88 | < 0.001 |  | 0.81 | 0.70-0.92 | 0.001 |
| >100k | 0.67 | 0.59-0.76 | < 0.001 |  | 0.73 | 0.64-0.83 | < 0.001 |
| Primary site lower lobe (reference) |  |  |  |  |  |  |  |
| Main bronchus | 1.19 | 1.09-1.32 | < 0.001 |  |  |  |  |
| Middle lobe | 0.81 | 0.74-0.90 | < 0.001 |  | 0.88 | 0.79-0.97 | 0.013 |
| Overlapping lesion | 1.12 | 0.92-1.33 | 0.271 |  |  |  |  |
| Upper lobe | 0.95 | 0.91-1.00 | < 0.001 |  | 0.93 | 0.89-0.98 | < 0.001 |
| Tumor Size <45 mm | 0.78 | 0.76-0.82 | < 0.001 |  | 0.83 | 0.80-0.87 | < 0.001 |
| Histology type Adenocarcinoma (reference) |  |  |  |  |  |  |  |
| Squamous cell carcinoma | 1.61 | 1.51-1.72 | < 0.001 |  | 1.39 | 1.30-1.48 | < 0.001 |
| Large cell carcinoma | 1.36 | 1.18-1.56 | < 0.001 |  | 1.34 | 1.17-1.55 | < 0.001 |
| Non-small cell carcinoma | 1.73 | 1.60-1.87 | < 0.001 |  | 1.51 | 1.39-1.63 | < 0.001 |
| Small cell carcinoma | 1.45 | 1.38-1.54 | < 0.001 |  | 1.56 | 1.46-1.66 | < 0.001 |
| Differentiated Moderate (reference) |  |  |  |  |  |  |  |
| Poor | 1.30 | 1.08-1.56 | < 0.001 |  |  |  |  |
| Undifferentiated | 1.26 | 0.91-1.76 | 0.170 |  |  |  |  |
| Well | 0.58 | 0.33-1.03 | 0.061 |  |  |  |  |
| Unknown | 1.53 | 1.31-1.80 | < 0.001 |  |  |  |  |
| Grade stage I (reference) |  |  |  |  |  |  |  |
| II | 1.06 | 0.87-1.32 | 0.536 |  |  |  |  |
| III | 1.32 | 1.08-1.62 | < 0.01 |  |  |  |  |
| IV | 1.36 | 1.08-1.72 | < 0.01 |  |  |  |  |
| Unknown | 1.04 | 0.85-1.27 | 0.686 |  |  |  |  |
| T stage T1 (reference) |  |  |  |  |  |  |  |
| T2 | 1.01 | 0.88-1.04 | 0.732 |  | 1.14 | 1.06-1.26 | < 0.001 |
| T3 | 1.04 | 0.90-1.11 | 0.954 |  | 1.18 | 1.04-1.28 | 0.002 |
| T4 | 1.13 | 1.03-1.25 | < 0.001 |  | 1.54 | 1.25-1.78 | 0.011 |
| N stage N0 (reference) |  |  |  |  |  |  |  |
| N1 | 1.04 | 0.97-1.13 | 0.240 |  | 1.09 | 1.01-1.18 | 0.034 |
| N2 | 1.22 | 1.17-1.29 | < 0.001 |  | 1.28 | 1.21-1.35 | < 0.001 |
| N3 | 1.25 | 1.18-1.34 | < 0.001 |  | 1.43 | 1.34-1.53 | < 0.001 |
| Node positive Yes | 0.82 | 0.79-0.87 | < 0.001 |  | 0.90 | 0.86-0.95 | < 0.001 |
| Diagnose to therapy ≥13 days | 1.14 | 1.09-1.19 | < 0.001 |  | 1.37 | 1.30-1.44 | < 0.001 |
| Primary site surgery Yes | 0.49 | 0.43-0.56 | < 0.001 |  | 0.59 | 0.51-0.67 | < 0.001 |
| Radiotherapy Yes | 0.38 | 0.34-0.45 | < 0.001 |  | 0.62 | 0.54-0.71 | < 0.001 |
| Chemotherapy Yes | 0.44 | 0.42-0.46 | < 0.001 |  | 0.44 | 0.42-0.46 | < 0.001 |

1k = 1,000 USD
